# Live cyanobacteria produce photocurrent and hydrogen using both the respiratory and photosynthetic systems

**DOI:** 10.1038/s41467-018-04613-x

**Published:** 2018-06-04

**Authors:** Gadiel Saper, Dan Kallmann, Felipe Conzuelo, Fangyuan Zhao, Tünde N. Tóth, Varda Liveanu, Sagit Meir, Jedrzej Szymanski, Asaph Aharoni, Wolfgang Schuhmann, Avner Rothschild, Gadi Schuster, Noam Adir

**Affiliations:** 1 The Nancy & Stephen Grand Technion Energy Program (GTEP), Technion – Israel Institute of Technology, Technion City, 32000 Haifa Israel; 2 Analytical Chemistry – Center for Electrochemical Sciences (CES), Ruhr-Universität Bochum, Universitätsstr. 150, 44780 Bochum, Germany; 3 Schulich Faculty of Chemistry, Technion – Israel Institute of Technology, Technion City, 32000 Haifa Israel; 4 Faculty of Biology, Technion – Israel Institute of Technology, Technion City, 32000 Haifa Israel; 5 Department of Plant and Environmental Sciences, The Weizmann Institute of Science, Rehovot, Israel; 6 Leibniz Institute of Plant Genetics and Crop Research (IPK), Network Analysis and Modelling, OT Gatersleben, 06466 Seeland, Germany; 7 Department of Materials Science and Engineering, Technion – Israel Institute of Technology, Technion City, 32000 Haifa Israel

## Abstract

Oxygenic photosynthetic organisms perform solar energy conversion of water and CO_2_ to O_2_ and sugar at a broad range of wavelengths and light intensities. These cells also metabolize sugars using a respiratory system that functionally overlaps the photosynthetic apparatus. In this study, we describe the harvesting of photocurrent used for hydrogen production from live cyanobacteria. A non-harmful gentle physical treatment of the cyanobacterial cells enables light-driven electron transfer by an endogenous mediator to a graphite electrode in a bio-photoelectrochemical cell, without the addition of sacrificial electron donors or acceptors. We show that the photocurrent is derived from photosystem I and that the electrons originate from carbohydrates digested by the respiratory system. Finally, the current is utilized for hydrogen evolution on the cathode at a bias of 0.65 V. Taken together, we present a bio-photoelectrochemical system where live cyanobacteria produce stable photocurrent that can generate hydrogen.

## Abstract (web summary)

Biologically ### produced electrical currents and hydrogen are new energy sources. Here, the authors find that low presser microfluidizer treatment produced cyanobacterium that can utilize electrons from respiratory and photosynthesis to promote current and hydrogen generation, without the addition of exogenous electron mediators.

## Introduction

**Fig. 1:**
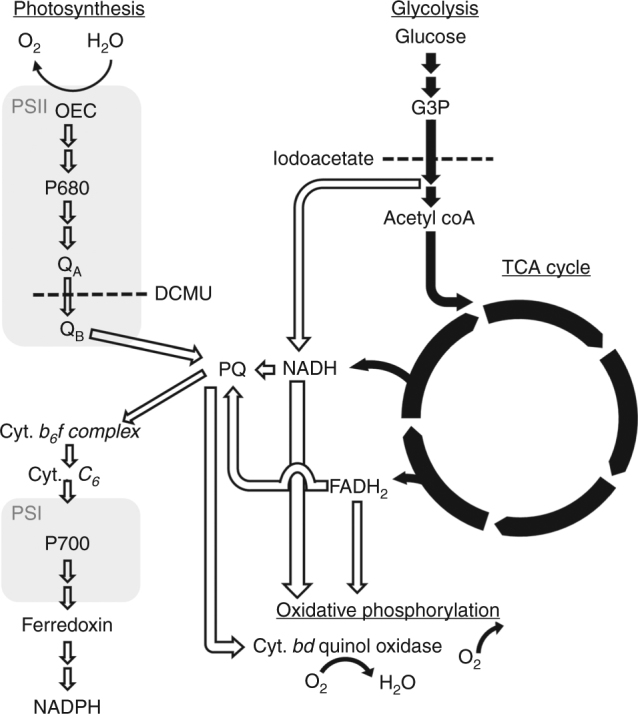
The electron flow of the photosynthetic process and respiration metabolism in cyanobacteria. The scheme was drawn based on the data described in references 12–21. Filled black arrows mark the respiration metabolism. Unfilled arrows indicate the photosynthetic and additional electron flow processes. Double arrows mark additional steps not shown for clarity. G3P is glyceraldehyde 3 phosphate, and TCA stands for tricarboxylic acid cycle. The membrane soluble plastoquinone (PQ) functions as an acceptor of residual electrons of the glycolysis and the TCA cycle, as well as an essential mediator of the photosynthetic electrons between PSII and the b_6_f complex. Dashed lines indicate the site of action of inhibitors of the photosynthetic or respiratory, used in this work

Cyanobacteria are a wide class of photosynthetic organisms that have been recently proposed as potential sources of materials (membranes, complexes) from which photoexcited electrons can be extracted to produce electrical current and renewable fuels^1–3^. Different species of cyanobacteria living in a wide range of habitats, were responsible for the original formation of the Earth’s oxygen enriched atmosphere, and continue to serve as a major source of primary production and oxygen evolution^4^. In cyanobacterial photosynthesis, the major photosynthetically relevant light absorbing pigment is chlorophyll (chl) *a*, which has maximal absorbance at 420–460 nm and 670–700 nm. The phycobilisome (PBS) antenna complex additionally absorbs between 550–680 nm. Photosystem I (PSI) also contains a minor antennae complex that has been shown to be red-shifted to ~710–730 nm^5–8^. The energy is efficiently transferred from the antennas to the Photosystem II (PSII) and PSI reaction centers (P_680_ and P_700_, respectively)^9–11^. In this light-driven process, electrons from water are sequentially transferred through a series of redox species to finally reduce NADP^+^ to be used for carbon fixation^12,13^ (Fig. 1). A proton-motive gradient is created between the thylakoid membrane lumen and the cytoplasm, resulting in proton-coupled electron transfer, which serves as the driving force for ATP production.

In the dark, cyanobacterial cells oxidize carbon sources via the respiratory system. For example, glucose is oxidized to acetyl CoA in the glycolytic process. Acetyl CoA is further oxidized in the tricarboxylic acid (TCA) cycle. These processes produce ATP, NADH, and FADH_2_. NADH and FADH_2_ are oxidized during oxidative phosphorylation to consume oxygen and produce CO_2_ and ATP^14–16^. Cyanobacteria lack the separation afforded by eukaryotic organelles, and it has been previously shown that electrons can flow between the photosynthetic and respiratory systems via redox active molecules^17^. For example, quinones from the photosynthetic plastoquinone (PQ) pool can oxidize NADH and FADH_2_, both products of glucose oxidation^17–21^ (Fig. 1).

Various attempts have been made to extract electrons from photosynthetic systems for the purpose of solar energy conversion (SEC)^19,22–29^. In some studies, isolated photosystems (PS) have been used within a bio-photoelectrochemical cell (BPEC) to produce electric power^30–34^. With isolated PS, an exogenous electron acceptor has to be used as a mediator (when utilizing PSI or bacterial reaction centers, an exogenous donor is also required) and the PS has to be fixed to the electrode, for instance by the presence of an immobilized mediator or a redox polymer^24,25,31,34^. In the case of PSII, water can serve as the electron donor, dependent on the stability of the oxygen evolving complex (OEC). One of the main problems with using isolated photosynthetic complexes for SEC is the relatively short lifetime of the biological components of the system, due to damage caused by radicals formed within the reaction centers^35,36^. Combined membrane systems have an evolutionary benefit for energy conversion that has recently been identified as a characteristic that could improve artificial photosynthetic SEC systems^35^. The use of living cells could mitigate this problem due to the presence of repair systems that can replace photo-damaged photosynthetic proteins. However, extracting electrons from living organisms is potentially more complicated as it may require an exogenous electron carrier (mediator) that can penetrate the cell wall and membrane, such as ferricyanide, cytochrome, or 2,6-dichloro-1,4-benzoquinone (DCBQ)^23,27,36–39^. To overcome this difficulty, cyanobacterial cells have been grown or dried on an electrode to extract an electric current by direct contact^1,28,40,41^. However, when such direct contact is utilized, the electric current is limited, as it will likely be generated from only a single layer of cells.

Here, we describe the construction of a BPEC in which live cyanobacteria produce a significant photocurrent that is derived from electrons of the respiratory and photosynthetic systems, without the need for exogenous electron donors or mediators. The resulting current was utilized for the production of molecular hydrogen on the cathode at a bias voltage of 0.65 V.

## Results and Discussion

### Production of a significant current by living cells

**Fig. 2:**
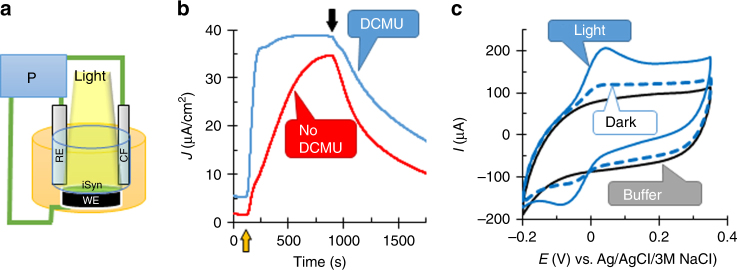
Gently treated *Synechocystis* cells generate an enhanced photocurrent using an endogenous redox mediator. **a** Schematic drawing of the bio photo-electrochemical apparatus with the iSyn settled on the working electrode (WE). The counter electrode (CE) is platinum, and the reference electrode (RE) an Ag/AgCl/3 M NaCl. All electrodes are connected to the potentiostat (P). **b** Chronoamperometric measurements at 150 mV (vs. Ag/AgCl/3 M NaCl) for iSyn in the presence (blue) or absence of DCMU (red). The yellow up arrow indicates light on (the initial 100 seconds show the dark current) and the black down arrow indicates light off. **c** Cyclic voltammograms of iSyn, illuminated (solid blue lines) or in the dark (dashed blue lines). Buffer lacking iSyn shows no redox activity (black solid line). Measurements recorded at a rate of 5 mV s^−1^

Cyanobacterial cells, *Synechocystis* sp. PCC 6803, were gently treated with a microfluidizer (herein called iSyn, see methods), and applied by gravity onto the graphite electrode. A platinum wire serves as the cathode while an Ag/AgCl/3 M NaCl electrode is used as a reference in all measurements (BPEC, Fig. 2a). When we measured current over time (chronoamperometry, CA), at an applied potential of 150 mV (vs. Ag/AgCl/3 M NaCl) (standard working potential obtained from the IV curve (Supplementary Fig. 1A), for all CA experiments unless mentioned otherwise), a dark current of 5 ± 1 µA cm^−2^ was obtained, probably directly from the respiratory system, as previously suggested^36,37^ (Fig. 2b, initial 100 seconds). Upon illumination, the current increased to the maximum stable current of 36 ± 4 µA cm^−2^. Accordingly, the net produced photocurrent was 31 ± 4 µA cm^−2^. Upon return to dark, there was a slow decline in the measured current. We noticed that the addition of the photosynthetic electron flow inhibitor, 3-(3,4-dichlorophenyl)-1,1-dimethylurea (DCMU), resulted in a significantly faster increase in the photocurrent, reaching the maximum value in about 3 min, as compared to about 13 min in the absence of DCMU (Fig. 2b). Thus, DCMU was used for all electrochemical measurements unless mentioned otherwise. Cyclic voltammetry (CV) of the iSyn indicated the presence of a redox active species with an anodic peak at 50 mV (vs. Ag/AgCl/3 M NaCl) and a cathodic peak at −70 mV (vs. Ag/AgCl/3 M NaCl). The anodic current was significantly increased during illumination to present a quasi-reversible voltammogram, suggesting a photo-induced catalytic reaction mediated by a redox species (Fig. 2c). Optimization experiments show that increasing the amount of cells increases both CV (Supplementary Fig. 1B) and CA (Supplementary Fig. 1C) values up to a plateau of 150–225 μg chlorophyll. Since no external mediating electron transfer molecules were added to the BPEC, we conclude that the source of the redox species identified in Fig. 2c is from within the Syn cells. The characterization of this mediator will be described in detail below.

**Fig. 3:**
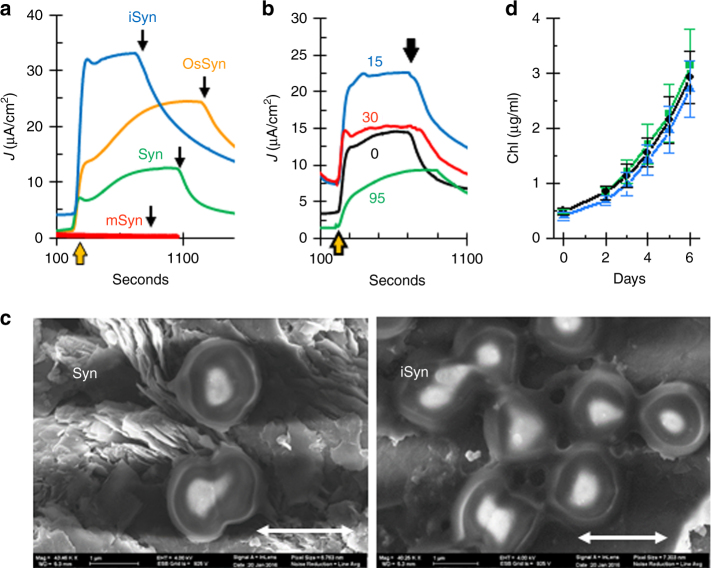
iSyn are living, modified, and clustered cells. **a** CA measurements of *Synechocystis* cells (Syn, green), cells undergone a mild osmotic shock treatment, (OsSyn, Orange), cells undergone a gentle pressure treatment (iSyn, blue), and thylakoid membranes (mSyn, red). **b** CA measurements of cell undergone treatment with microfluidizer at 0, 15, 30, and 95 psi, as indicated in the figure. **c** SEM image of iSyn (right) and Syn (left) on graphite. Scale bars are 2 μm. **d** Growth curve of Syn (green), iSyn (black) and iSyn that were illuminated for 30 min in the BEPC (blue) in liquid medium. The vertical lines show the standard deviation (*n *= 5)

We noticed that there was a negative correlation between the pressure used during the treatment performed on the cells and the measured current (Fig. 3a). The highest current was obtained when the lowest possible pressure of the microfluidizer was used (Fig. 3b). Lower currents were obtained when using untreated cells (Syn) or cells that were osmotically shocked (OsSyn). Membrane fragments obtained from cells that were completely disrupted at higher pressure with a French Pressure cell (mSyn) did not produce measureable currents (Fig. 3a). The microfluidizer treatment (iSyn) resulted with a higher anodic peak in the dark, compared to OsSyn and Syn (Supplementary Fig. 1D), whereas membrane fragments did not show any anodic peak (Supplementary Fig. 1E) indicating the absence of the redox species (see methods for all preparations). iSyn appear to retain their cellular structure and remain clumped together as visualized by scanning electron microscopy (SEM) and florescent microscopy (Fig. 3c, Supplementary Fig. 2A, respectively). To further investigate the state of the iSyn preparation, we utilized confocal fluorescence microscopy. Syn, OsSyn, and iSyn appeared in the microscope to retain PBS functional integrity. Treatment of the same cells using higher pressures (mSyn) produces aggregated membranous material, lacking the PBSs (Supplementary Fig. 2A-C), which are soluble and spontaneously released from the membranes^9^. The major difference between the Syn and iSyn is that the latter are clumped together in large clusters (Fig. 3c, Supplementary Fig. 2A). In the OsSyn preparation, cells are seen to form smaller clusters of two or three cells with the same appearance of fusion (Supplementary Fig. 2A). To determined cell viability, we prepared cells as described above, and illuminated them for 30 min in the BPEC under standard conditions. We then removed the iSyn from the BPEC by simple washing. The growth rate of the iSyn was then compared to the growth rates of either untreated Syn or non-illuminated iSyn in new growth media. All cells had similar growth rates (Fig. 3d and Supplementary Fig. 3a). To further determine cell viability, we performed a colony formation test (Supplementary Fig. 3B). We applied an amount of Syn, iSyn, or membranes (mSyn) containing the same amount of chlorophyll onto a growth plate, and followed the growth of the colonies. Syn and iSyn grow at a similar rate, 364 ± 98 and 280 ± 68 colonies per femtograms of *Chl a* to Syn and iSyn, respectively, whereas mSyn contains much smaller amount of living cells (11 ± 3). These results indicate that the application of mild pressure did not harm the ability of *Synechocystis* cells to multiply and divide. iSyn were also shown to photosynthetically evolve O_2_ at a similar rate to Syn cells (O_2_ evolution of 392 ± 53, 464 ± 60 nmol O_2_ µg chl^−1^ h^−1^ for iSyn and Syn, respectively). We thus suggest that iSyn are living, yet modified and/or clustered cells and that the change that occurs to the cells does not modify the biological energy transducing systems.

### The combined activity of two pathways generates the current

**Fig. 4:**
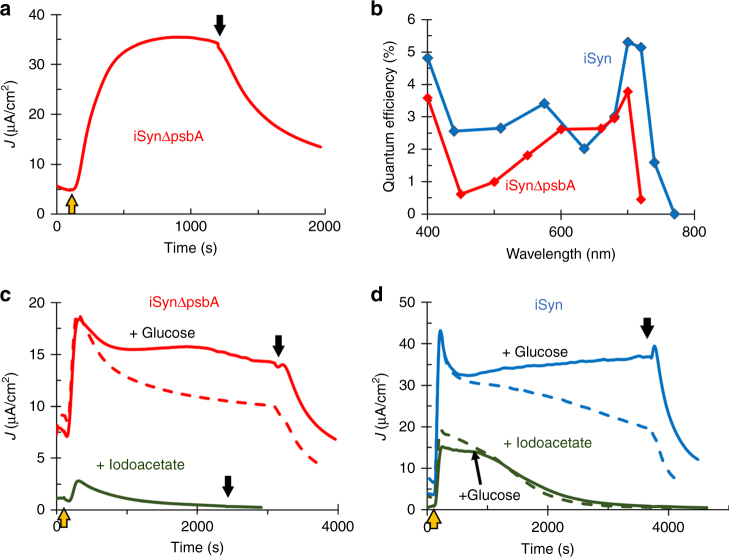
iSyn photocurrent to the BPEC involves both photosynthetic and respiratory systems. **a** CA measurements for iSynΔpsbA. **b** Action spectra: the obtained photocurrent was normalized to the photon energy input (external quantum efficiency) at different wavelengths. The external quantum efficiency of iSyn is shown in blue and that of iSynΔpsbA in red. A new measurement with fresh cells was used for each wavelength. **c** CA measurements for iSynΔpsbA without (red) and with (green) the addition of iodoacetate and in the presence (full line) or absence (dashed line) of glucose. **d** CA measurements for iSyn without (blue) and with (green) the addition of iodoacetate or in the presence (full line) or absence (dashed line) of glucose in the buffer

The photocurrent in the presence of DCMU could be explained by two alternative pathways. In the first one, the electrons are derived from water oxidation and are transferred to putative endogenous mediator(s) prior to Q_B_, before the binding site of DCMU at Q_B_, similar to the electron transfer from PSII to silicomolybdate^42,43^. The second possibility is that DCMU inhibits the activity of PSII, including the water oxidation complex. In such a case, a light-driven electron source must be located further downstream of the Q_B_ site, and could involve the PQ pool, cytochrome *b*_*6*_*f*, and finally PSI, as the site of photoactivity. A stable photocurrent that is not a result of PSII activity can only exist if there is an alternative source of electrons to PSI. Such a possible source could be the respiratory system that is linked to the photosynthetic apparatus in cyanobacteria^14,17–21^ (Fig. 1). To differentiate between these possibilities we repeated the electrochemical experiments with a non-autotrophic mutant of *Synechocystis* sp. PCC 6803, where the *psbA* genes (encoding the D1 protein) have been deleted, thus leading to a non-functional PSII (ΔpsbA)^43,44^ (Supplementary Fig. 4A). CA measurements displayed a photocurrent that is similar to that obtained with iSyn (Fig. 4a). Moreover, the iSynΔpsbA cells look similar to iSyn cells (Supplementary Fig. 4B) and require the same preparation for optimal photocurrent as iSyn does (Supplementary Fig. 4B-D). The iSynΔpsbA cells also contain the same redox mediator (Supplementary Fig. 4D). Another proof of the lack of involvement of PSII in the electron transfer pathway in the BPEC was to inactivate the OEC of PSII by incubation with Tris buffer. In the Tris treated iSyn similar photocurrents were obtained (Supplementary Fig. 5A-B). Taken together these results indicate that the electron path is from the respiratory system, through PSI and to the electrode.

To further support the notion that the photosystem deriving the photocurrent is PSI and not PSII, we measured the action spectrum of the photocurrent production. To this end, we measured the photocurrent as a function of the wavelength and calculated the external quantum efficiency (EQE) at each wavelength (Fig. 4b). The EQE in both iSyn and iSynΔpsbA was evident at all wavelengths of visible light with an increase at 700 nm suggesting the importance of the participation of PSI in the activity. Together, these results demonstrated that PSI and not PSII is the photosystem that drives the photocurrent when iSyn cells are illuminated in the BPEC.

As already indicated above, the lack of the water oxidation activity of PSII in the iSynΔpsbA indicates that there must be an alternative electron donor. Studies suggest that electron transfer between the respiratory system and the photosynthetic system may occur via the plastoquinone (PQ) pool^14,18^. To investigate this, we performed experiments in the presence of glucose. Glucose is known to be uptaken by *Synechocystis* cells, providing energy for growth in the dark or for growth of non-photosynthetic mutants^43,44^. Addition of glucose significantly extended the period of the production of the highest current (Fig. 4c). Furthermore, iodoacetate, which blocks the respiratory path at the site of glyceraldehyde-3-phosphosphate dehydrogenase activity (Fig. 1), significantly inhibited the photocurrent (Fig. 4c), supporting the suggestion that the respiratory system is the main electron donor.

The above measurements support our hypothesis that under these conditions a respiratory-PSI electron transfer pathway exists in the ΔpsbA mutant. In a fashion similar to that seen for iSynΔpsbA, iSyn also exhibits extended photocurrent lifetime in the presence of glucose (Fig. 4d and Supplementary Fig. 5C-D), and the photocurrent is decreased in the presence of iodoacetate (Fig. 4d), reaffirming that the major electron source is the respiratory system. Together, the results so far uncover an electron transfer pathway in which electrons derived from the respiratory pathway reduce PSI that can further photo-reduce the electrode in the gently treated *Synechocystis* live cells.

### The mediator is a small diffusive soluble molecule

**Fig. 5:**
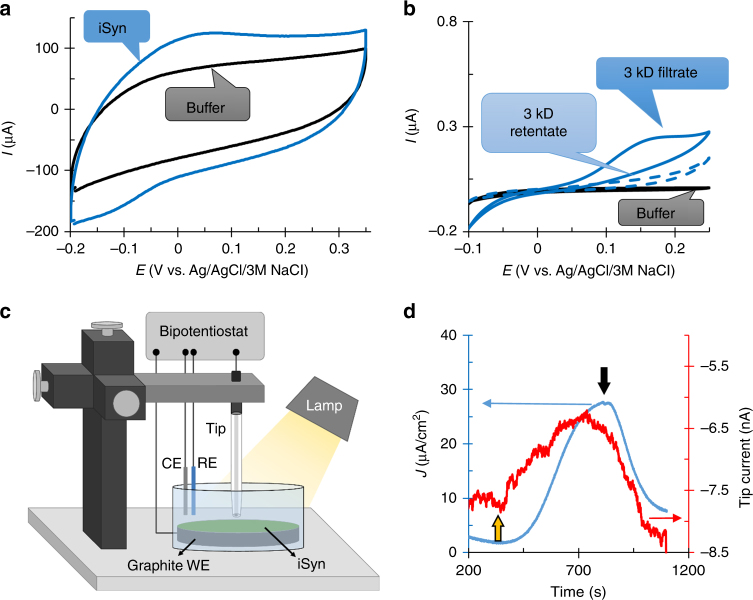
iSyn electron transfer to the BPEC is via a diffusive endogenous mediator. **a** CV in the light for buffer (black) and the iSyn (blue) separated by a 3 kD dialysis membrane on the graphite electrode. **b** CV of supernatant fraction from centrifuged iSyn cells separated by filtration through 3 kD cut-off centricon. Filtrate (solid blue line), retentate (dashed blue), and buffer (black). **c** Illustration of the scanning electrochemical microscopy setup. iSyn settled on the working electrode (WE) electrode. The counter electrode (CE) is platinum, and the reference electrode (RE) a Ag/AgCl/3 M KCl. The tip is a carbon-based microelectrode with a mixture of bilirubin oxidase (BOD) embedded in an Os-complex modified polymer matrix. All electrodes are connected to the bipotentiostat. **d** CA of the iSyn (blue) and the BOD tip current (red) measuring the oxidation of the mediator at a distance of 30 µm from the graphite electrode

Electrochemical experiments show a slow increase and slower decline in the photocurrent (Fig. 2b). Moreover, the potential difference between the anodic and cathodic peaks in the voltammogram (Fig. 2c) suggests that the current in the BPEC is mediated by a diffusive soluble molecule. Cyanobacterial cells contain both small redox active proteins, as well as metabolites that undergo redox reactions. We introduced a single layer synthetic membrane with a nominal molecular weight cut-off of 3 kD between the iSyn and the electrode (Fig. 5a) and performed CV measurements under light. These show the same anodic and cathodic peaks in the presence of iSyn as without the membrane, suggesting the mediator can diffuse through a 3-kD membrane and is thus most likely not a protein.

To further isolate the mediator, iSyn were illuminated followed by centrifugation. The supernatant was then filtered through a 3 kD ultrafiltration device and CV measurements were performed on both the filtrate and retentate. These measurements show that only the filtrate contains a redox active species while the retentate has no redox active component (Fig. 5b). This indicates that the mediator is a water-soluble molecule that passes through the 3-kD filter. Similar experiments performed on dark exposed iSyn (Supplementary Fig. 6A) showed the presence of a redox peak but with a smaller area, suggesting that illumination increases the release of the mediator from the cells. In addition, the redox behavior of the mediator is inactivated after 10 min incubation at 60 °C (Supplementary Fig. 6B). We suggest that the redox molecules could be a soluble quinone, a flavonoid^44,45^ or a small peptide that is temperature sensitive.

Scanning electrochemical microscopy (SECM) was used to exclude the possible release of O_2_ under irradiation and proved the existence of a soluble mediator in accordance with the CA measurements. SECM measures the local current on a microelectrode tip precisely positioned above the evaluated sample. SECM can be used to measure local electrochemical reactions over an evaluated sample or to efficiently collect reaction products with the tip placed in close proximity to the analyzed surface, for example, it was previously used to measure oxygen production by n-type semiconductor-based photo-anodes for water splitting^45,46^. The SECM experiment was performed by placing the tip microelectrode above the center of the cells at ~30 µm from the graphite electrode surface with iSyn deposited on top (Fig. 5c). As SECM probe we used a carbon-based microelectrode modified with bilirubin oxidase (BOD) embedded in an Os-complex modified polymer matrix^47,48^. The electrode was polarized at 0 mV (vs. Ag/AgCl/3 M KCl) for the steady-state reduction of O_2_. To decrease the background current related with the reduction of O_2_ naturally present in solution, Ar was bubbled into the buffer for 15 min before the measurement and continuously, gently, bubbled during the measurement. The obtained results in the SECM measurements clearly demonstrate the absence of any cathodic current related to the collection of O_2_ evolved from the sample. Furthermore, a considerable shift in the recorded current at the microelectrode toward more positive values was observed under irradiation and following a clear correlation between the sample current and the tip current under dark and light conditions (Fig. 5d). The shift in current at the SECM probe can then be attributed to the oxidation of the soluble mediator at the tip microelectrode that is polarized at a more negative potential than the redox peak observed for the mediator in previous experiments (Fig. 2c). Further measurements at a tip potential of −200 mV (vs. Ag/AgCl/3 M KCl) indicate a reduction of the mediator in the light (Supplementary Fig. 6C). SECM measurements confirmed that the mediator diffuses from the iSyn through the solution to the tip. Thus, we can conclude that there is a water-soluble electron carrier that mediates between the iSyn and the electrode.

Finally, in an attempt to identify the electron carrier mediator if it is a metabolite, we carried out a metabolic profiling^49,50^ of Syn, iSyn, and iSyn that were illuminated in the BEPC. No significant change between the different samples profile was detected, suggesting that the gentle treatment does not cause a significant change in the amount of a metabolite but rather the release of the mediator to the medium or the outrace of the cells (Supplementary Fig. 7 and Supplementary Table 2).

### DCMU has a unique effect in the iSyn BPEC

In the presence of DCMU, the maximum current is reached faster and the current is slightly enhanced (Fig. 2b, Supplementary Fig. 8A,B). However, the photocurrent lifetime is prolonged by the presence of added glucose in either the presence or absence of DCMU (Supplementary Fig. 8C), suggesting that DCMU affects the electron flow but not the stability of the photocurrent. Counter-intuitively, the presence of DCMU was found to be crucial for the photocurrent production by the iSynΔpsbA, since in the absence of DCMU the photocurrent drops to 14% of the photocurrent obtained in the presence of DCMU (Supplementary Fig. 8B,D). Thus, even though iSynΔpsbA lacks the well-studied PSII-binding-site of DCMU, the addition of the herbicide has a dramatic effect and actually was essential for the production of the 20–30 µA cm^−2^ current obtained with iSynΔpsbA even without DCMU (Supplementary Fig. 8D). The effect of DCMU in the iSynΔpsbA strongly indicates that DCMU has secondary binding sites with *Synechocystis* cells that have not yet been characterized. Moreover, DCMU increase the release of the mediator (Supplementary Fig. 8E) and as a results increases the photocurrent and decreases the time it takes to reach the maximum photocurrent (Fig. 2b, Supplementary Fig. 8C). Our results suggest that DCMU affects the production of the photocurrent in both iSyn and iSynΔpsbA at secondary site(s) by either inhibiting forward electron flow within the respiratory system, or by enhancing the release of the mediator.

### Hydrogen is produced in the BPEC

**Fig. 6:**
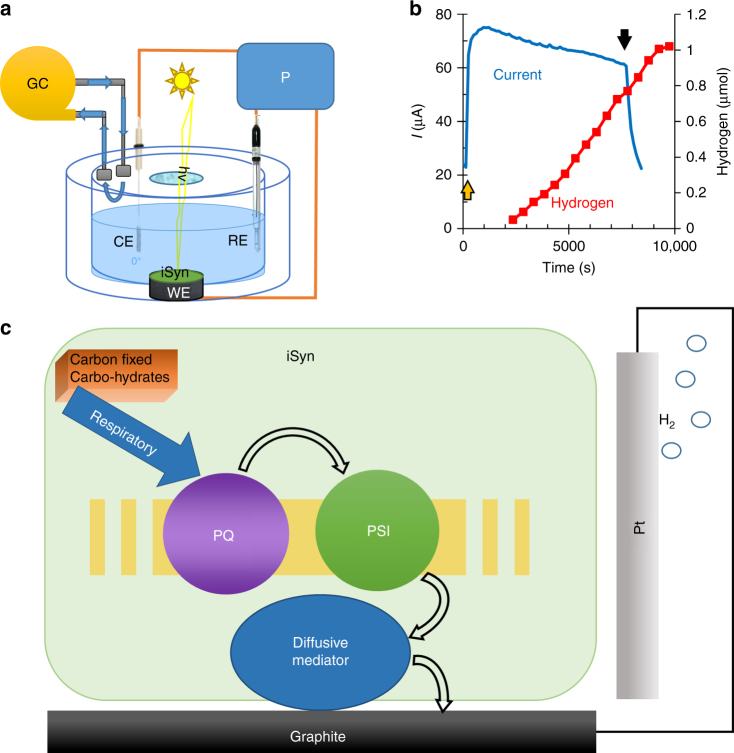
Hydrogen is evolved on the cathode at a lower voltage than in water electrolysis. **a** Schematic drawing of the working setup for the hydrogen evolution measurements. The CA was measured at 50 mV (vs. Ag/AgCl/3 M NaCl) which corresponds to a voltage of 650 mV between the anode and cathode. **b** Simultaneous CA measurement of photocurrent (blue) and GC measurement of hydrogen production (red) measured as a function of time for the iSyn at 50 mV (vs. Ag/AgCl/3 M NaCl). **c** Schematic drawing of the electron flow from carbohydrates (internal or external) via the respiratory, light-driven activity of PSI, and an endogenous diffusive mediator to form hydrogen gas. The multiple electron transfer steps between the PQ and PSI are shown for simplicity as multiple curved arrows

As no external redox components were added to the BPEC, we presumed that protons are reduced to molecular hydrogen at the Pt cathode. To measure hydrogen production, we built a gas-tight BPEC connected directly to a gas-chromatograph (GC) (Fig. 6a). The gas actively diffuses via ducts and is pumped into the GC, hence there is a short delay in the gas measurement. Figure 6b shows the H_2_(g) production over time measured using this configuration applying a voltage of 650 mV between the anode and cathode. The lack of H_2_(g) production in the absence of light and/or iSyn (Supplementary Table 1) indicates a correlation between the light-current and the H_2_ production. During the 2 h of illumination an average of 3.5 µmol H_2(g)_ mg Chl^−1^ h^−1^ is produced at a Faradaic efficiency of ca. 36%. The low Faradaic efficiency is most likely the result of molecular oxygen in the buffer, indicated by higher Faradaic efficiency in the absence of oxygen (Supplementary Fig. 9), or the mediator reduced on the cathode in addition to proton reduction.

In this study, we show light-driven current and hydrogen production with mildly treated live *Synechocystis* sp. PCC 6803 on a graphite electrode. The simple treatment makes the electron transfer possible from the cells to the electrode. This is the first time CV measurements indicate the existence of an endogenous mediator that can exit the cells’ membranes in mildly treated live cyanobacteria^50,51^. The electron extraction, by the intrinsic mediator, is photo-driven by PSI (Fig. 6c) that is reduced by the respiratory system, explaining the enhancing effect of glucose addition. In the absence of externally added glucose, the current originates from the internal carbohydrate pool produced during the growth phase. Whether PSI reduces the mediator directly or through other components of the photosynthetic electron transfer chain has not yet been ascertained. It is important to note that current is obtained from non-treated cells as well, however at much lower rates. This reiterates that the role of the treatment is to augment the amount of released mediator, and not the inducing of any modification to either of the two biological systems. During the review process of this article, a manuscript suggesting mediator dependent electron transfer in cyanobacteria was published.^52,53^ In this work, porous translucent electrodes were used in a BEPC cell containing *Synechocystis* cells to obtain electric currents that were higher than obtained in nonporous electrodes but significantly lower than reported in this work using iSyn and graphite electrode.

The use of iSyn instead of thylakoids may have the potential to utilize physiological repair mechanisms during the operation of the BPEC. This system, based on live *Synechocystis* and an endogenous mediator, can further be used to study extraction of electrons from live cells and with some future modifications can be used to generate photocurrent for a long time by cell rejuvenation.

In the recent past, utilization of biological systems for environmentally clean energy production has been offered as an attractive alternative to the use of fossil fuels. Our results presented here show that Cyanobacteria offer a unique opportunity to fully utilize the potential of biological energy conversion systems. We have shown here that in these live cells, electrons released during the reactions of the respiratory system can be “pumped” back to higher energy by the photosynthetic system, enabling both sustained current and the reduction of hydrogen at lower added bias. Indeed, coupled with other innovative uses of the solar spectrum^24^ we believe that the cyanobacterial system can serve a true paradigm for clean bio-energy production.

## Methods

### Preparation of mildly treated cells of *Synechocystis*

Cells of a late log phase culture (OD_750_ = 1.0–1.5) that was grown as previously described in Larom et al.^38,39^ were centrifuged for 10 min at 15,200× *g* in a SLA-1500 rotor and resuspended in Buffer A (20 mM Tris pH 8, 400 mM NaCl and 15 mM MgCl_2_). The cells were then passed twice through a microfluidizer (*Microfluidics Corporation*) under a pressure of 10–15 psi. The cells were centrifuged for 20 min at 27,660× *g* and the cell pellet was resuspended in 100 mM phosphate buffer (pH 6). The final concentration of chl *a* was determined in 80% (v/v) acetone according to Arnon^53,54^. The final chlorophyll concentration of all samples was 0.6–1.3 mg chl ml^−1^.

### Preparation of all other *Synechocystis* samples

Thylakoid membranes (mSyn) were isolated in a fashion similar to the iSyn preparation; however, a French pressure cell (*Sim Aminco* Spectronic Instruments) operating at 1500 psi was used instead of the microfluidizer, followed by centrifugation for 2 min at 600× *g* in order to pellet unbroken cells and 20 min centrifugation at 27,660× *g* to pellet the thylakoids. For mSynΔpsbA preparation, we used the microfluidizer at high pressure of 90 psi circulating for 28 pulses. Untreated cells (Syn) were pelleted and resuspended in phosphat buffer (pH 6). Osmotic shocked cells (OsSyn) were resuspended in buffer A and pelleted after 20 min and resuspended in phosphate buffer (pH 6). The cells treated with different pressures were prepared similar to iSyn, but under higher pressure in the microfluidizer step. The final concentration of all samples was 0.6–1.3 mg chl ml^−1^.

### Electrochemical measurements

Electrochemical measurements were performed either at the Solar Simulation Lab (Viterbi Faculty of Electrical Engineering, Technion) using a Zennium electrochemical workstation (*Zahner Elektrik*) and a solar simulator light source (*Oriel Sol3A*), or at the Hydrogen Lab (Grand Technion Energy Program) with an nStat multichannel potentiostat (*Ivium*) and a Xenon lamp solar simulator (*Abet*). For some measurements, we used a Palmsens3 (*Palmsens*) potentiostat and A1 light line (*Sciencetech*). All measurements were carried out in a three electrodes configuration under the illumination of one solar unit (1SU). Graphite (*Graphite Store, pn#bl001230*) was used as a working electrode, a Pt electrode (*ALS*) served as the counter electrode and a Ag/AgCl/3 M NaCl as a reference electrode (*ALS*). For each measurement, a sample containing 150 µg of chl *a* was placed on the 1.8 cm diameter graphite electrode. The sample was not stirred or replaced during the measurement. The medium was phosphate buffer (100 mM phosphate, pH 6) containing 100 mM NaCl. When indicated, 150 µM of DCMU was added. For the ΔpsbA mutant, chlorophyll amount of 50 µg was used. For the relevant electrochemical measurements, 6 mM of D-glucose in the absence or presence of 5 mM iodoacetic acid (sodium salt) were added.

### Quantum efficiency measurements

The EQE of the BPEC containing iSyn (150 µg chl) was measured at a potential of 150 mV (vs. Ag/AgCl/3 M NaCl). The iSyn cells were illuminated with monochromatic light obtained by coupling a white light source to a monochromator (Cornerstone CS260) with a spectral bandwidth of 10 nm. Fresh cells were used in each measurement, and the maximum photocurrent was recorded. The photon flux was obtained from the light intensity measured by a power meter (918D High Performance Photodiode Sensor), and the EQE was calculated according to equation 1:

1 $${\rm EQE} = \frac{{{\mathrm{Maximum}}\,{\mathrm{photocurrent}}\,/\,q}}{{{\mathrm{Photon}}\,{\mathrm{flux}}}}$$

where *q* is the electron charge.

When analyzing iSynΔpsbA we used 50 µg chl for each measurement and the bandwidth was 20 nm.

### Oxygen evolution measurements

For measurement the photosynthetic activity by analyzing the rate of oxygen evolution, the cells (10 µg chl a) were placed in phosphate buffer (pH 6) in a final volume of 1 ml. A volume of 3 mM 2,6-dichloro-1,4-benzoquinone (DCBQ) was used as an electron acceptor and oxygen evolution was determined using a Clark-type electrode (*Hansatech*). Following preincubation in the dark for 2 min, the cells were illuminated with a white light halogen projector lamp (*MRC*) for 1 min and the increase in oxygen concentration was digitally measured.

### Gas chromatography experiments

Hydrogen measurements were performed in the Hydrogen Lab (Grad Technion Energy Program) using an Agilent 7890 A GC system (*Agilent technologies*) with a molsieve 5 Å column, 10 ft*1/8”*2 mm (*Agilent technologies*) to determine H_2_ production. Gas from the BPEC was continuously pumped to the GC. The applied potential on the working electrode was 50 mV (vs. Ag/AgCl/3 M NaCl). The bias between the anode and cathode measured by a voltmeter (m832, *Mastech*) was 650 mV. Calibration was performed by measuring known amounts of hydrogen directly injected to the GC.

### Scanning electron microscopy

The morphology of cellular samples was characterized by high-resolution scanning electron microscopy (SEM) (Ultra PLUS, *Zeiss*). Further experimental details are found in the figure legend.

### Purification of the mediator

iSyn cells, correspond to 400 µg chlorophyll from the stock solution, were illuminated for 5 min (without DCMU) in a 1.5 ml tube and then centrifuged (5417 R, *Eppendorf*) for 1 min at max speed. The supernatant was collected, the pellet was resuspended in 250 µl Phosphate buffer, centrifuged again, and the supernatant was collected. Filtration was performed using 3 kD (Vivaspin 500, *Sartorius Stedim biotech*) and centrifugation (5417 R, *Eppendorf*) for 1 h at 14,000× *g*. Heat inactivation was performed in a block heater (SKS *Biomedical instruments*) at 60 °C for 10 min.

### Scanning electrochemical microscopy

SECM experiments were performed using a Ag/AgCl/3 M KCl double junction reference electrode, a Pt cylindrical mesh as counter electrode, and a BOD modified carbon microelectrode as electrochemical probe. The BOD modified tip was fabricated following a previously reported procedure^54,55^ by modifying a carbon-based microelectrode with a mixture of BOD embedded in an Os-complex modified polymer matrix^47,48^. The SECM setup is schematically shown in Fig. 5c and is based on a previously described system^46,47^ consisting of three stepmotor driven micrometer screws (*Owis*) for positioning of the SECM tip in *x*-, *y*-, and *z*-directions, a bipotentiostat (PGU-BI 100, *Jaissle*), and a control software programmed using Visual Basic 6.0. A 150 W Hg–Xe lamp (LC8, Hamamatsu photonics) was used for irradiation of the sample at an incident light intensity of 55 mW cm^−2^ (visible light). The sample was placed in a Teflon SECM cell sealed with an O-ring exposing an area of about 0.385 cm^2^. The tip was positioned in close proximity (~30 µm) to the analyzed sample by recording z-approach curves in air-saturated buffer before adding the iSyn. The BOD modified tip was polarized at 0 or - 200 mV (vs. Ag/AgCl/3 M KCl). During the measurement, a given potential of 150 mV (vs. Ag/AgCl/3 M KCl) was applied to the sample. Both the tip and sample current were recorded during light and dark periods. For the SECM measurements, we used frozen/thawed iSyn cells. After preparation of the cells as described above, dimethyl sulfoxide was added to the cells, prior to flash-freezing in liquid nitrogen. iSyn was later thawed on ice and used for the SECM experiments.

Procedures for PBS spectroscopic measurements, confocal imaging, Tris treatment, colony formation test, and metablomics profiling were detailed in Supplementary Methods.

### Data availability

All data presented in this paper can be obtained from the corresponding authors upon reasonable request.

## Electronic supplementary material

Supplementary Information
