# Supplementary material for: Live cyanobacteria produce photocurrent and hydrogen using both the respiratory and photosynthetic systems
Source: Nat Commun. 2018 Jun 4;9:2168. doi: 10.1038/s41467-018-04613-x (PMC5986869; doi:10.1038/s41467-018-04613-x)
Supplement: Supplementary file 1 — Supplementary Information [file 41467_2018_4613_MOESM1_ESM.docx]

**Live cyanobacteria produce photocurrent and hydrogen using both the respiratory and photosynthetic systems**

Saper and Kallmann *et al.*

**Supplementary Methods**

**PBS spectroscopic measurements**

The iSyn and supernatants from the samples preparation step (FP, microfluidizer) after the last centrifuge were measured on a Cary 100Bio spectrophotometer.

**Confocal imaging**

Confocal microscope LSM 510 META or LSM 710 (*Zeiss*) was used for fluorescence imaging (X40 magnitude, water immersed). For phycobilisome excitation a 633 nm HeNe laser was used and a filter of >650 nm. Chl *a* was excited by 458 nm multiline Aragon laser and same filter was used to select the fluorescence photons. ZEN 2012 (*Zeiss*) software was used for image processing.

**Tris treatment**

iSyn where placed in a solution of 1 M 2-Amino-2-(hydroxymethyl)propane-1,3-diol (tris) PH=9.2 for one hour at 4 ◦C. The solution was than centrifuged at 13000 RPM at 4 ◦C with a Heraeus MegaFuge 16R (Thermo Scientific) and the cells were suspended in 100 mM phosphate buffer.

**Colony formation test**

Samples were produced as described previously. Each sample was diluted with fresh BG11 to final concentrations of 333, 33, 16.5, 3.3, and 1.6 pg chl *a*. From each diluted sample 3 μl were dropped on an Agar-BG11 plate. The plate was incubated for ~20 days.

**Metablomics profiling**

Three samples of each treatment: Syn (Ctr 1-3), iSyn (iSyn 1-3) and iSyn following 30 min illumination in the BEPC iSynL were collected and subjected to metabolite profiling analysis ^1^. The final data matrix have been generated by substituting the values of overloaded peaks by their respective measurements in split samples corrected for the dilution factor, basing on internal standard measurements. Subsequently data have been double normalized: first to the internal standard ribitol, and second to the total ion count per sample. Finally, all values have been scaled between 0 and 100.

**
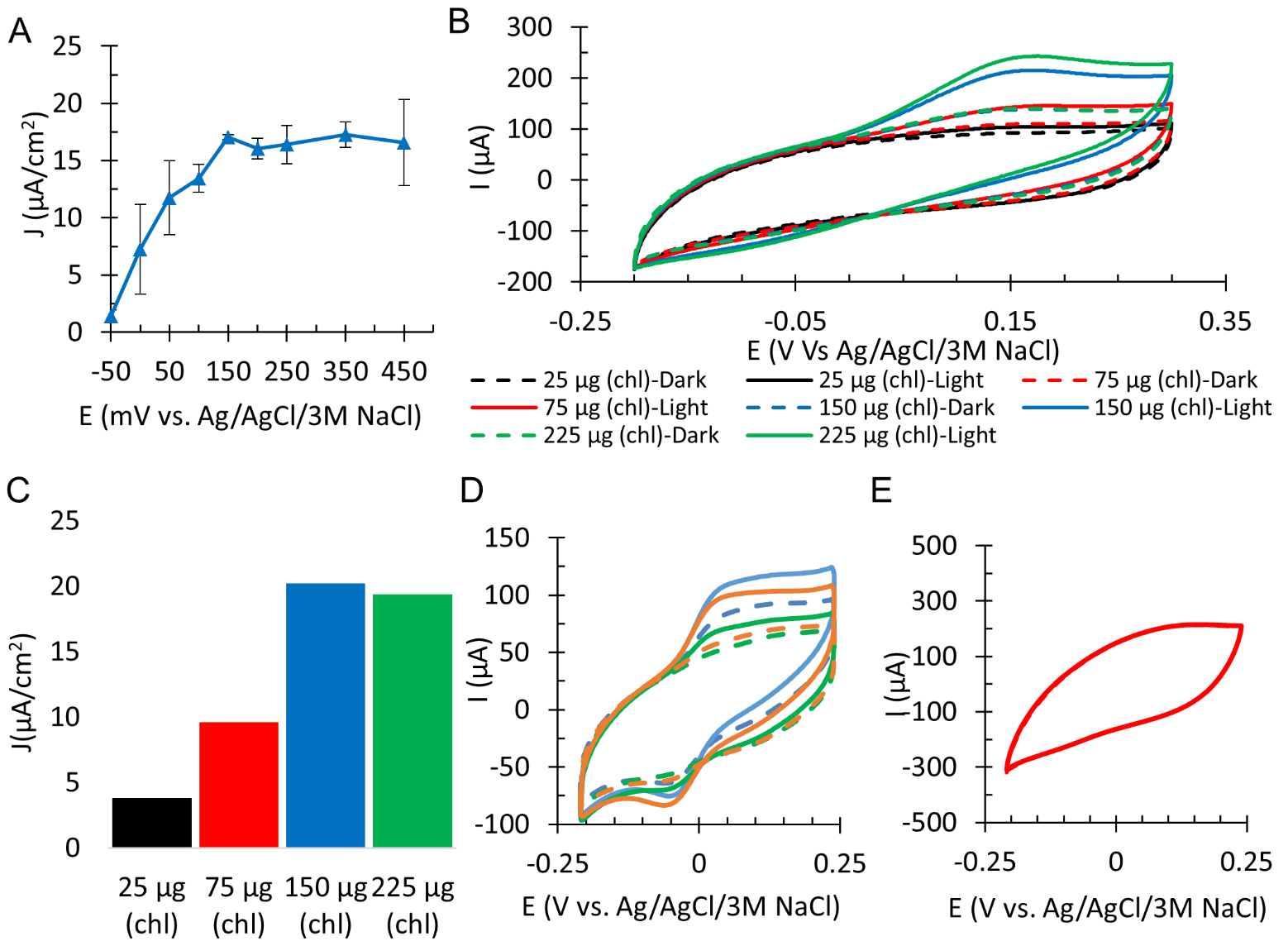
**

**Supplementary Figure 1. Gentle treatment of *Synechocystis* sp. PCC 6803 produces maximum photocurrent at 150 mV (vs. Ag/AgCl/3M NaCl) in the presence of DCMU.** (A) Current as a function of potential. The currents were measured in CA mode. Error bars represent the standard deviation of three independent experiments. The maximum photocurrent is obtained at potentials >150 mV (vs. Ag/AgCl/3M NaCl). (B) CV for the 25 µg (*chl*) (black), 75 µg (*chl*) (red), 150 µg (*chl*) (blue) and 225 µg (*chl*) (green) of iSyn in the dark (dashed line) or in the light (full line). (C) Photocurrent obtained from CA for 25 µg (*chl*) (black), 75 µg (*chl*) (red), 150 µg (*chl*) (blue) and 225 µg (*chl*) (green) of iSyn. (D) CV for the Syn (green), OsSyn (orange) and iSyn (blue) either under illumination (full lines) or in the dark (dashed lines). (E) CV for membranes obtained by French pressure cell disruption (mSyn). All scan rates were performed at 10 mV/sec.


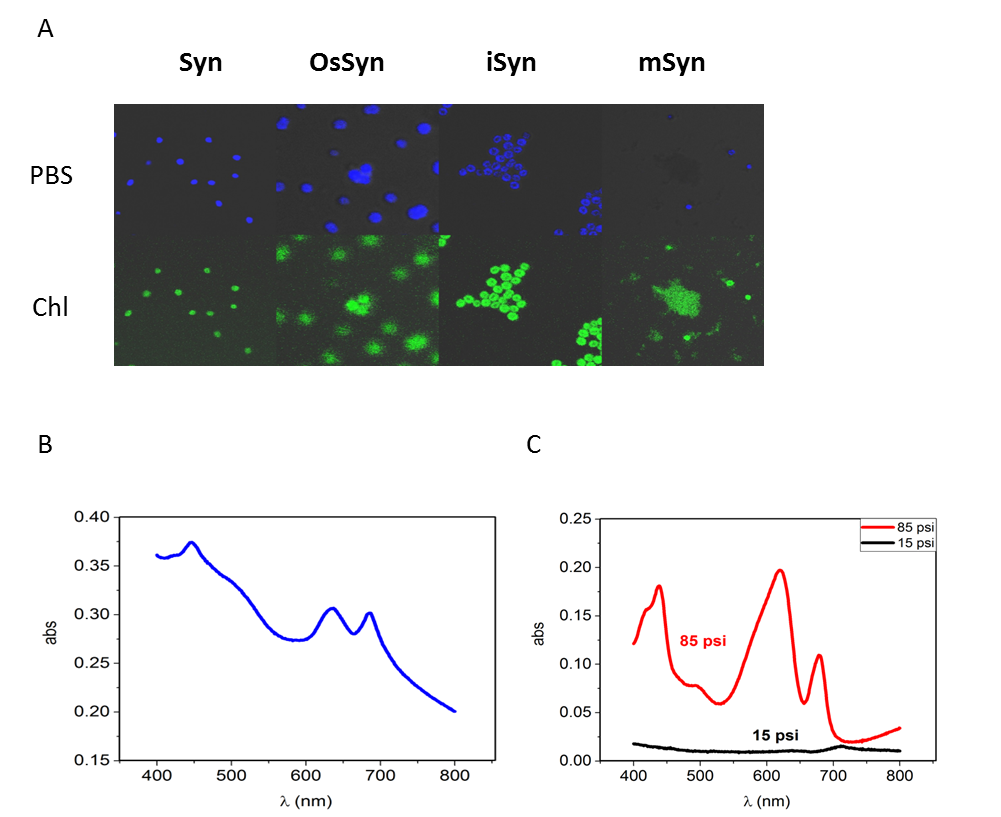


**Supplemental Figure 2.** **iSyn contain phycobilisomes (PBS) and maintain cellular structure.** (A) Confocal microscopic images of the various treated cyanobacteria cells. Top line: the artificially colored blue represents excitation at 633 nm and emission at >650 nm showing the presence of active PBS energy transfer to reaction centers^2^. Bottom line: Artificially colored green represents excitation at 458 nm (chlorophyll *a* in reaction centers) and emission at >650 nm^3^. Images of Syn, iSyn and OsSyn indicate emission from chlorophyll, regardless of excitation energy source. Images of the thylakoid membranes (mSyn) show emission only when excited at 458 nm, indicating the loss of functional PBS (which is released during cell disruption). (B) iSyn absorbance spectra indicate the existence of the PBS (at 600-650nm) not released during microfluidizer treatment. (C) Absorbance spectra obtained from supernatant of centrifuged iSyn (15 psi, black) or *Synechocystis* cells treatment at 85 psi with the microfluidizer (85 psi, red). High-pressure treatment disrupts the cells and releases the phycobilisomes.

Syn

1 X10 X20 X100 X200

iSyn

mSyn

B

A

**Supplemental Figure 3.** **iSyn are living and multiplying cells.** (A) Growth curve of Syn (green), iSyn (black) and iSyn that were illuminated for 30 min in the BEPC (blue), in liquid medium was examined by the absorption at 750nm. The vertical lines show the standard deviation values (n=5). (B) Colony formation test for Syn, iSyn and photosynthetic membranes (mSyn). Syn, iSyn and mSyn were plated at serial dilutions on agar plate containing growth medium. The equivalent amount of cells or membranes containing 1 ng of chlorophyll was plated in the lanes marked 1.


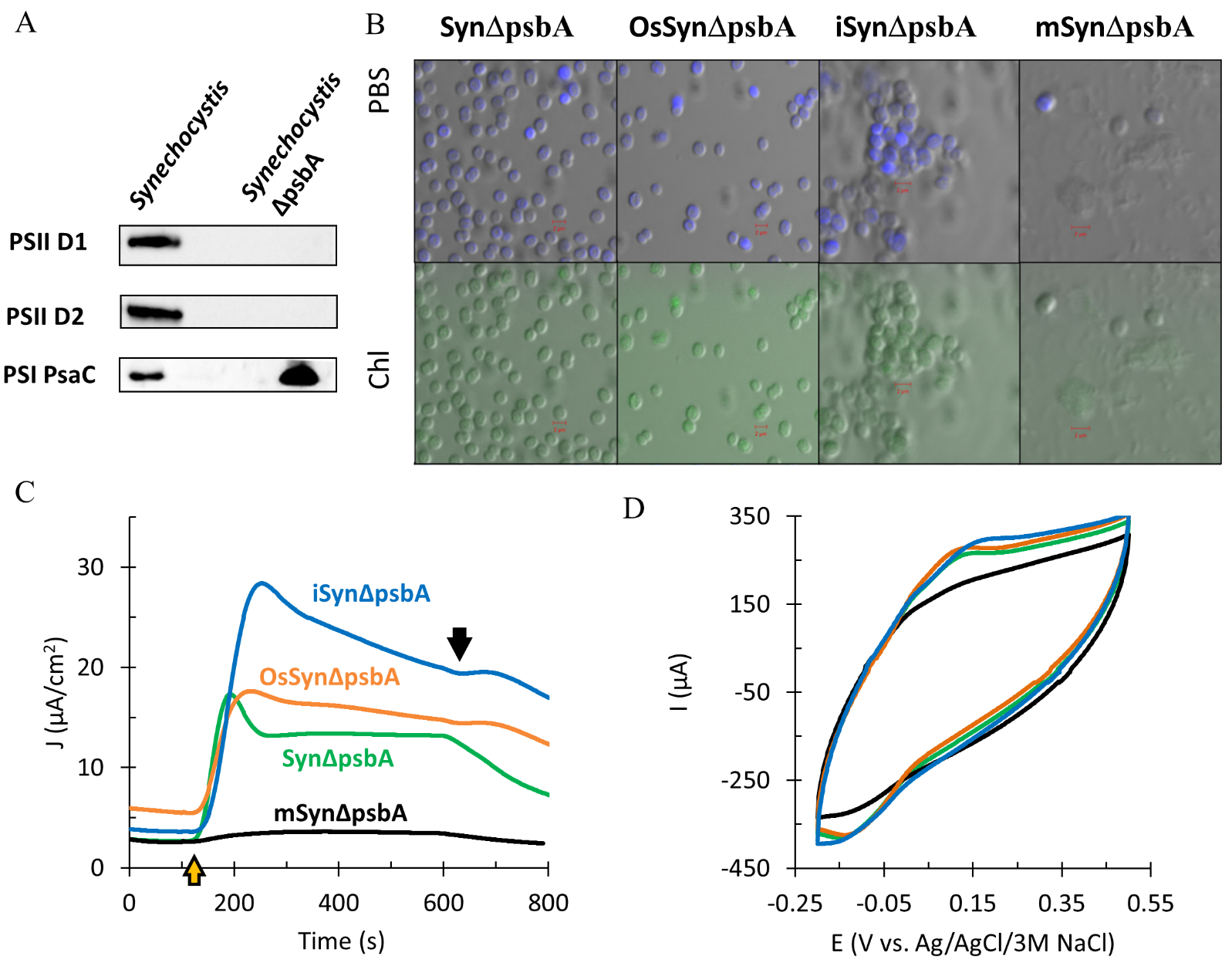


**Supplemental Figure 4. Mild treatment of the iSynΔpsbA maintains cellular structure and generates photocurrents.** (A) Western blot analysis of *Synechocystis* sp.PCC 6803 and *Synechocystis* sp.PCC 6803-ΔpsbA protein extract, using specific antibodies against photosystem II and I subunits (Agrisera, catalogue #AS05084 and #AS10939, respectively). Each lane was loaded with the equivalent amount of proteins related to 5 µg Chl., immunoblotted to membrane and decorated with specific antibodies for the D1 or D2 PSII reaction center proteins (products of the *psbA* and *psbD* genes, respectively), or the *psaC* gene product of PSI. *Synechocystis* sp.PCC 6803-ΔpsbA contains undetectable D1 and D2 PSII subunits, indicating the lack of this complex. (B) Confocal microscopic imaging of the various treated *Synechocystis* sp.PCC 6803-ΔpsbA, colored as described in Fig. S2A. The iSynΔpsbA cells are clustered together and seem to have a non-rounded shaped compared to the untreated SynΔpsbA. The mSynΔpsbA (passed through a microfluizider at 95 psi for 28 pulses and then centrifuged and re-suspended in phosphate buffer) are from disrupted cells that have lost their cell wall integrity and do not have functional phycobilisome. (C) CA measurements for ΔpsbA cyanobacteria cells with different treatments: SynΔpsbA (green), OsSynΔpsbA (orange), iSynΔpsbA (blue) and mSynΔpsbA (black). (D) CV for the SynΔpsbA (green), OsSynΔpsbA (orange), iSynΔpsbA (blue) and mSynΔpsbA (black) in the light suggests, that the treatment does not change the redox potential and that the endogenous mediator is the same electron shuttle in all preparations with very little mediator in the membranes. Scan rate: 20 mV/sec.


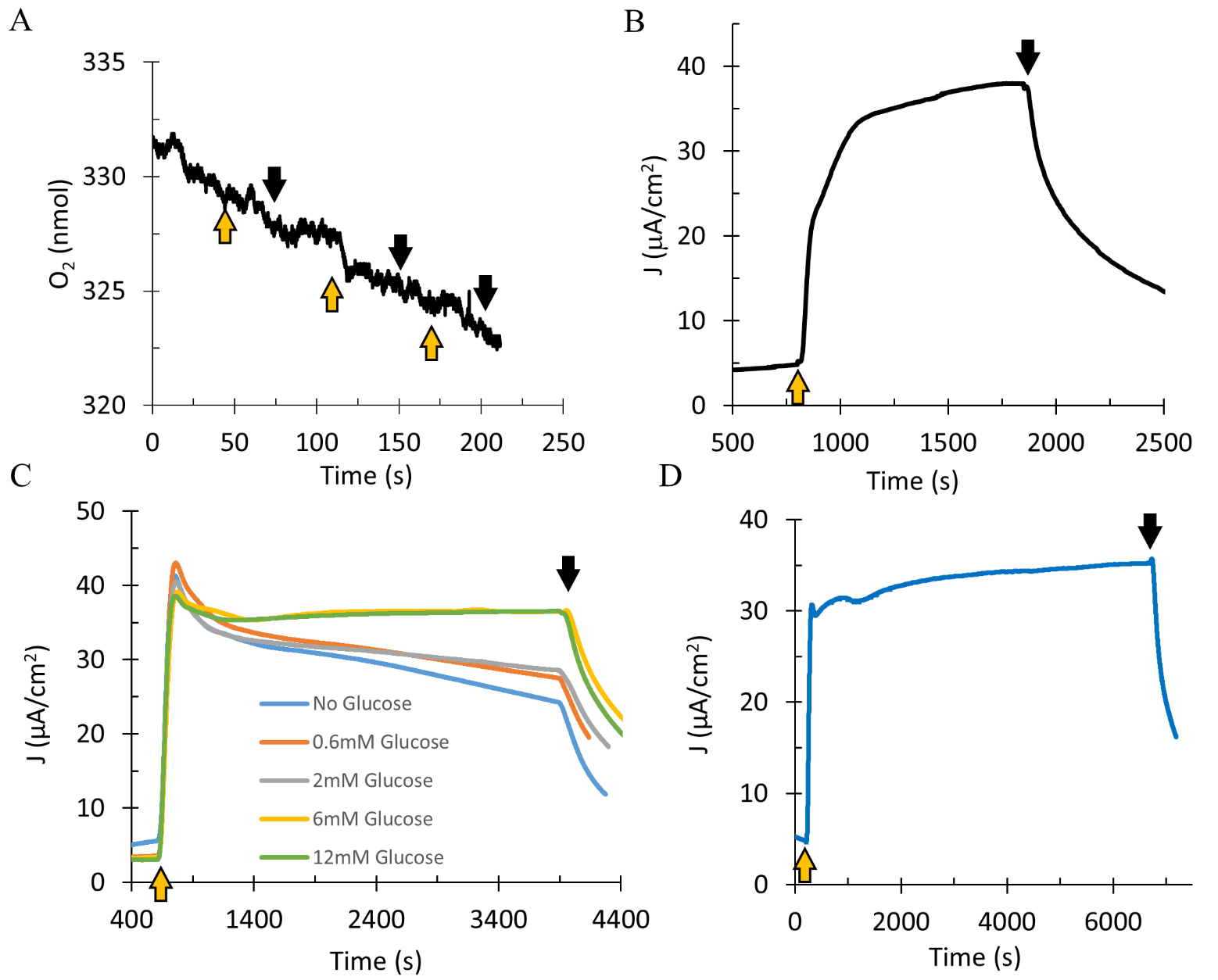


**Supplemental Figure 5. Photo-current originates from the respiratory system.** (A) Oxygen measured with a Clark electrode with DCBQ as an electron acceptor for the iSyn after treatment with 1 M 2-Amino-2-(hydroxymethyl)propane-1,3-diol (tris) PH=9.2 for 1 hour indicates no photo-increase in oxygen evolution, suggesting inactivation of the OEC. The Yellow up arrows indicates light on and black down arrows indicates light off. (B) CA measurements (at 50 mV vs. Ag/AgCl/3M NaCl) for iSyn after treatment for one hour in 1 M Tris. The treatment with Tris removes the OEC from PSII. (C) CA with increasing concentration of glucose. (D) Typical CA for the iSyn with the addition of 6 mM glucose indicates that glucose sustains the photocurrent for over 1.5 hours without a decrease of the photocurrent.


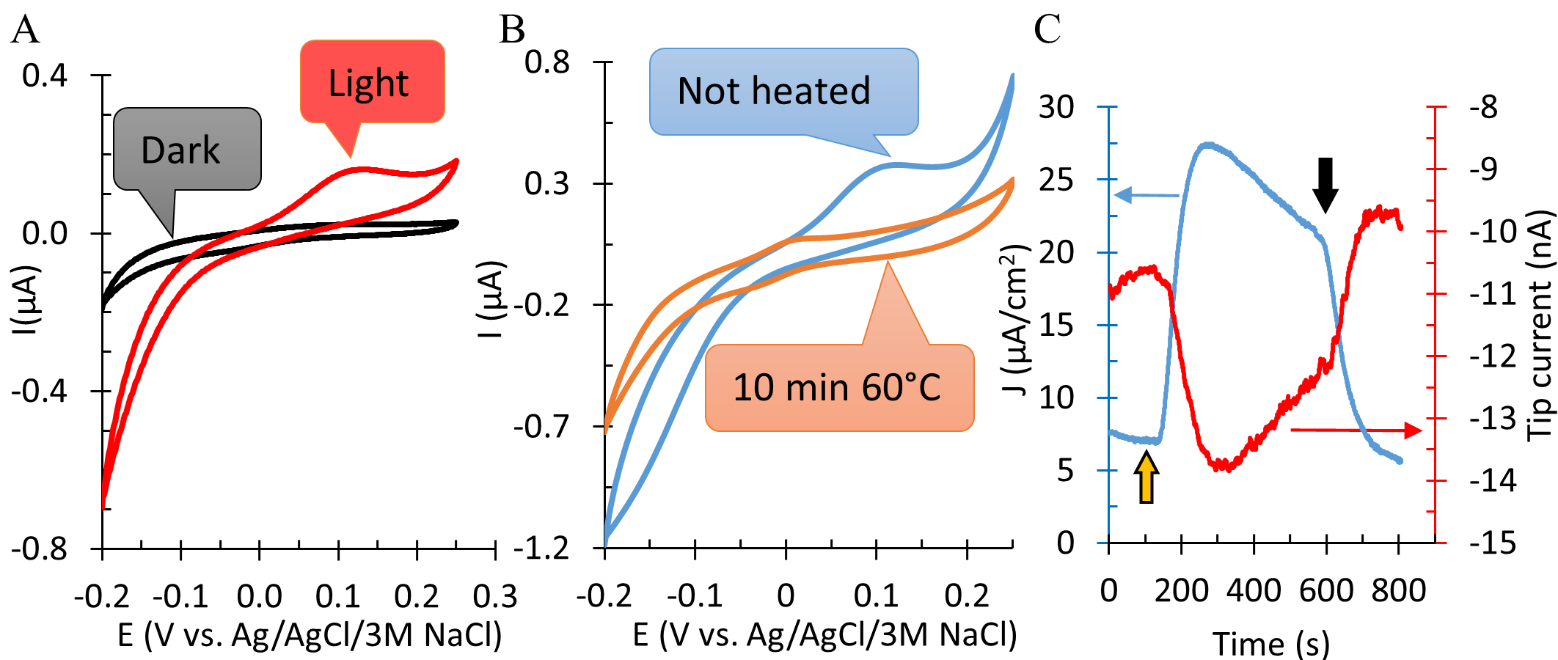


**Supplemental Figure 6. Characterization of the endogenous mediator**. (A) CV of the filtrate of separation through 3 kD cut of membrane, after 5 min incubation of the iSyn under 1 SU illumination (red) or in the dark (black) before the filtration. The average anodic peak area is 9.71·10^-4^ ±6.48·10^-4^, 875·10^-3^ ±5.05·10^-4^ µA·V for dark and light respectively, suggesting that light induces the release of the mediator. (B) CV of the 3 kD filtrate after incubation in 60 ^◦^C for 10 min (orange) compared to room temperature for 10 min (blue). (C) CA of the iSyn (blue) and the BOD tip current (red) measuring the reduction of the mediator at -200 mV vs. Ag/AgCl/3M KCl at a distance of 30 µm from the graphite electrode.

B

A

**Supplemental Figure 7. Metabolic profiling of Syn and iSyn did not reveal significant changes and the soluble mediator.**  Three samples of each treatment: Syn (Ctr 1-3), iSyn (iSyn 1-3) and iSyn following 30 min illumination in the BEPC iSynL (1-3) were collected and subjected to metabolite profiling analysis as described in ref. 3. Quantification and statistical analysis were done as described in this reference. The final data matrix have been generated by substituting the values of overloaded peaks by their respective measurements in split samples corrected for the dilution factor, basing on internal standard measurements. Subsequently data have been double normalized: first to the internal standard ribitol, and second to the total ion count per sample. Finally, all values have been scaled between 0 and 100. Clustering presented in the heatmap (A) was done on row-wise normalized log-scaled matrix using Euclidean distance and complete linkage agglomeration method. Metabolites marked with bold font were identified as exhibiting treatment related responses by the ANOVA analysis (Table S2) which are however below statistical significance. The PCA has been performed on log values, with unit variance scaling and mean-centered matrix and singular value decomposition method (B).


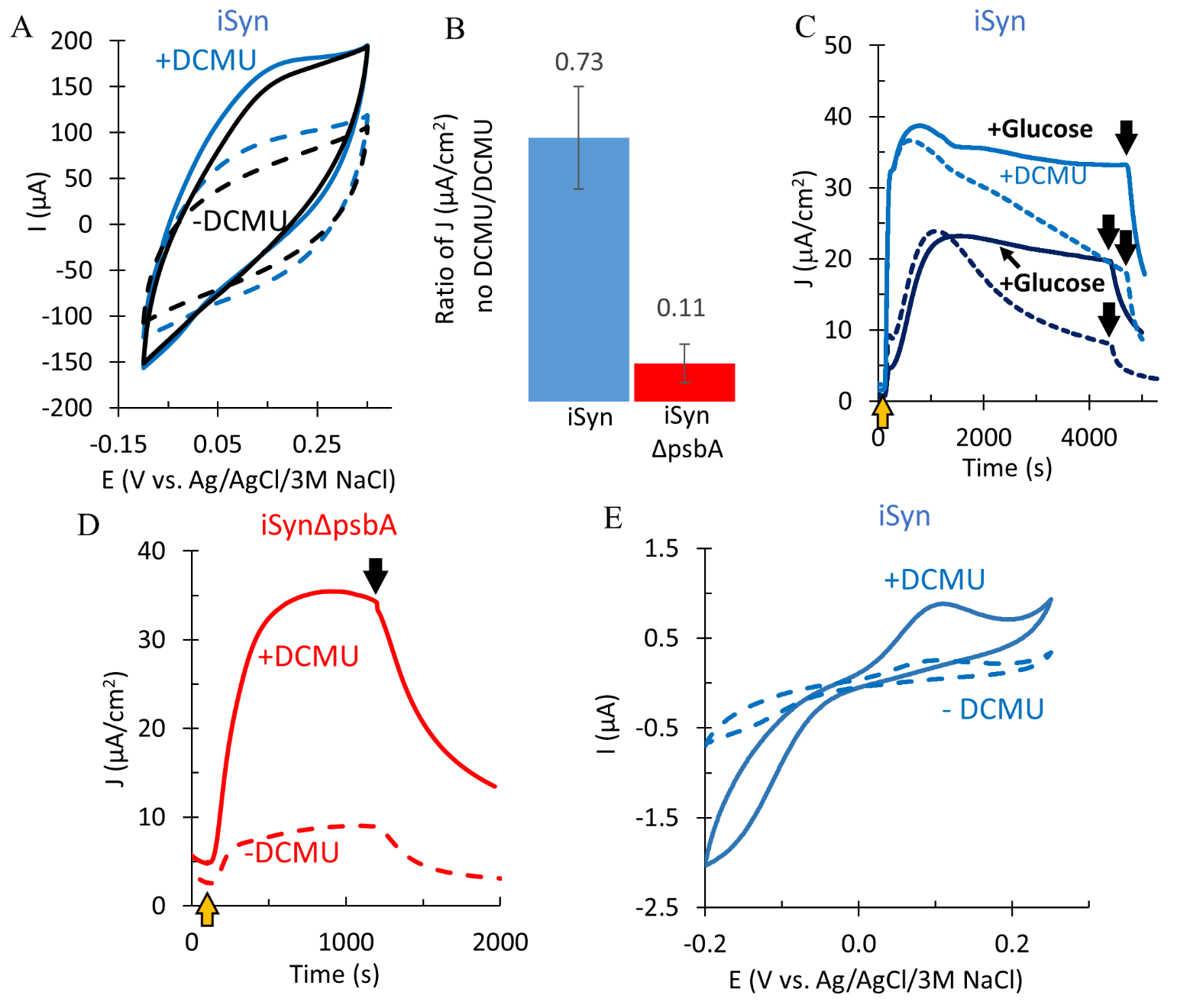


**Supplemental Figure 8.** **DCMU effect on the current.** (A) CV of iSyn with (blue) and without (black) 150 µM DCMU indicate photo-catalysis (light, solid lines; dark, dashed lines) at similar potential, suggesting the same mediator is active with or without DCMU. Scan rate 10 mV/sec. (B) The ratio of the photocurrent obtained without DCMU divided by the photocurrent obtained in the presence of DCMU for iSyn (blue) and iSynΔpsbA (red), averaged over 3 measurements. (C) CA measurements for the iSyn in the presence (blue) or absence (black) of DCMU, and in the presence (full line) or absence of (dashed line) of glucose. (D) CA measurements for iSynΔpsbA with (blue) or without (red) DCMU. (E) CV of the 3 kD filtrate, after 5 min incubation of the iSyn in the dark in the presence (full line) or absence (dashed line) of DCMU before the filtration.


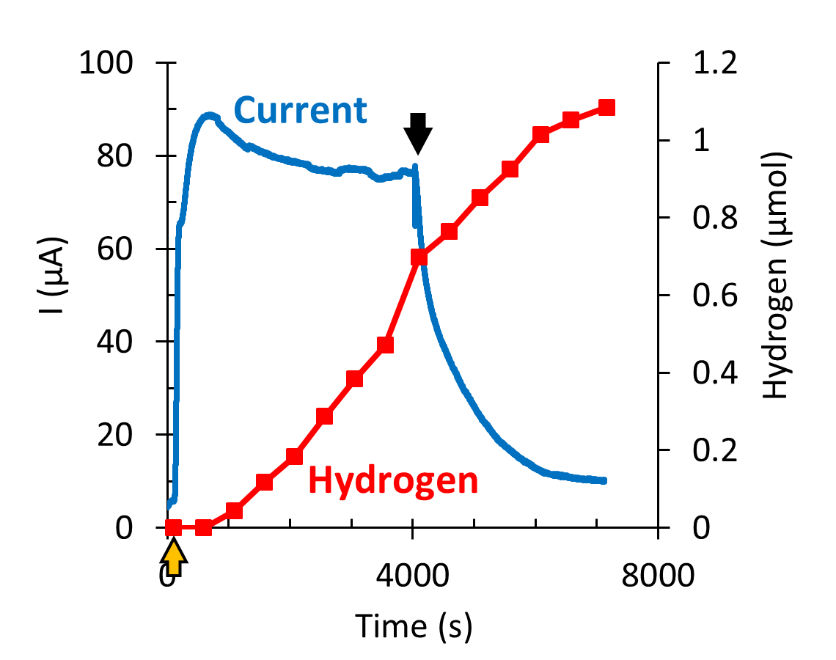


**Supplemental Figure 9.** **Hydrogen production in the absence of Oxygen.** Simultaneous CA measurement of photocurrent (blue) and GC measurement of hydrogen production (red) measured as a function of time for the iSyn at 50 mV (vs. Ag/AgCl/3M NaCl) after bubbling the gas tight BPEC with N_2_ for ~ 30min. The Faradic efficiency was 55%.

**Supplemental Table 1. H_2(g)_ production in the BPEC**

|  | iSyn +light  + bias*^a^* | No iSyn +light  + bias*^a^* | iSyn +light  No bias | iSyn in the dark  + bias*^a^* |
| --- | --- | --- | --- | --- |
| µmol H_2_ | 1.02 | 0 | 0 | 0.05> |

*^a^* Bias added between the anode and cathode was 650 mV.

**Supplemental Table 2. Results of the ANOVA analysis of the metabolic profile data**

|  | F value | p-value | FDR-adjusted p-value |
| --- | --- | --- | --- |
| Glycine | 2.806038769 | 0.137950719 | 0.238563258 |
| Acetic acid | 0.37295417 | 0.703610713 | 0.436521883 |
| Benzoic acid | 0.067822891 | 0.935131988 | 0.507292596 |
| Phosphoric acid | 0.590598098 | 0.583261586 | 0.391055067 |
| Glycerol | 0.18955712 | 0.832094424 | 0.478121653 |
| Isoleucine | 1.770017757 | 0.248773306 | 0.260444158 |
| Proline | 1.268187269 | 0.347242893 | 0.280532877 |
| Glycine | 1.202486684 | 0.363784948 | 0.285987505 |
| Succinic acid | 5.859756921 | 0.038823827 | 0.199781473 |
| Serine | 1.545653023 | 0.287458343 | 0.264508261 |
| Threonine | 2.943231642 | 0.128616235 | 0.237254113 |
| Beta Alanine | 5.189842166 | 0.049151544 | 0.209752287 |
| Malic acid | 13.24663027 | 0.006296133 | 0.108881341 |
| Aspartic acid | 2.890421982 | 0.132106607 | 0.237763573 |
| Pyroglutamic acid | 1.4518662 | 0.306011293 | 0.266127369 |
| N.Acetylglutamic acid | 3.65324236 | 0.091677705 | 0.229746823 |
| Cysteine | 1.199859735 | 0.364468002 | 0.286370709 |
| X2 ketoglutaric acid | 8.341209406 | 0.018509115 | 0.160042471 |
| Glutamic.acid | 1.744666839 | 0.252782262 | 0.260916595 |
| Phenylalanine | 0.529364501 | 0.614149673 | 0.403410031 |
| Glyceric acid 3P | 2.855258634 | 0.134501 | 0.238098961 |
| Citric acid derivative | 3.568602679 | 0.095267504 | 0.230704606 |
| Tetradecanoic acid | 1.46090639 | 0.304154632 | 0.265973386 |
| Fructose | 0.955119523 | 0.436400186 | 0.324546487 |
| Glucose | 0.938624289 | 0.441906205 | 0.327301036 |
| Lysine | 1.442432244 | 0.307964975 | 0.266287584 |
| Gluconic acid | 0.538534328 | 0.609387477 | 0.401537986 |
| Hexadecanoic acid | 1.79490165 | 0.244920227 | 0.259977203 |
| Myo-inositol | 0.88243411 | 0.461372264 | 0.336861947 |
| Octadecanoic.acid | 1.636182878 | 0.270945591 | 0.262900433 |
| Hexadecanoic acid propyl ester | 1.983873487 | 0.218103551 | 0.25632885 |
| Sucrose | 0.206033125 | 0.819331682 | 0.474266197 |
| Octadecanoic acid propyl ester | 1.994876547 | 0.216665363 | 0.256110961 |

**Supplementary References**

1. Treves, H. et al. Metabolic flexibility underpins growth capabilities of the fastest growing alga. *Curr. Biol.* 27, 2559–2567.e3 (2017).

2. Gantt, E. Phycobilisomes. *Annu. Rev. Plant Physiol.* **32,** 327–347 (1981).

3. Murata, N., Nishimura, M. & Takamiya, A. Fluorescence of chlorophyll in photosynthetic systems. 3. Emission and action spectra of fluorescence--three emission bands of chlorophyll a and the energy transfer between two pigment systems. *Biochim. Biophys. Acta* **126,** 234–43 (1966).
